# (Acetato-κ*O*)(aqua-κ*O*)(2-{bis­[(3,5-dimethyl-1*H*-pyrazol-1-yl-κ*N* ^2^)methyl]amino-κ*N*}ethanol-κ*O*)nickel(II) perchlorate monohydrate

**DOI:** 10.1107/S1600536812007970

**Published:** 2012-03-10

**Authors:** Jia Zhou, Mouhai Shu

**Affiliations:** a State Key Laboratory of Metal Matrix Composites, School of Chemistry and Chemical Engineering, Shanghai Jiao Tong University, Shanghai 200240, People’s Republic of China

## Abstract

In the structure of the title complex, [Ni(CH_3_CO_2_)(C_14_H_23_N_5_O)(H_2_O)]ClO_4_·H_2_O, the Ni^II^ centre has a distorted octa­hedral environment defined by one O and three N atoms derived from the tetra­dentate ligand, and two O atoms, one from a water mol­ecule and the other from an acetate anion. The mol­ecules are connected into a three-dimensional architecture by O—H⋯O hydrogen bonds. The perchlorate anion is disordered over two positions; the major component has a site-occupancy factor of 0.525 (19).

## Related literature

For the preparation of the tripodal ligand, see: Malachowski *et al.* (1992 ▶). For background to hydrolytic enzymes, see: Koike *et al.* (1995 ▶); Lipscomb & Sträter (1996 ▶). For related structures, see: Shin *et al.* (2011 ▶); Sundaravel *et al.* (2011 ▶); Xia *et al.* (2001 ▶).
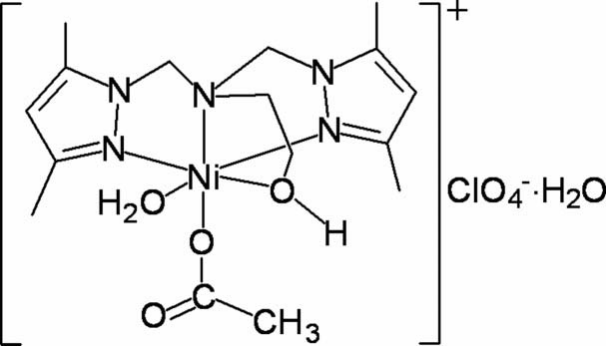


## Experimental

### 

#### Crystal data

[Ni(C_2_H_3_O_2_)(C_14_H_23_N_5_O)(H_2_O)]ClO_4_·H_2_O
*M* *_r_* = 530.61
Monoclinic, 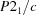

*a* = 9.6055 (11) Å
*b* = 9.9889 (11) Å
*c* = 24.258 (3) Å
β = 90.284 (2)°
*V* = 2327.5 (5) Å^3^
*Z* = 4
Mo *K*α radiation
μ = 1.00 mm^−1^
*T* = 293 K
0.43 × 0.37 × 0.21 mm

#### Data collection

Bruker APEX CCD diffractometer
Absorption correction: empirical (using intensity measurements) (*SADABS*; Sheldrick, 2003 ▶) *T*_min_ = 0.732, *T*_max_ = 1.000
13249 measured reflections
5057 independent reflections
2284 reflections with *I* > 2σ(*I*)
*R*_int_ = 0.082

#### Refinement

*R*[*F* ^2^ > 2σ(*F* ^2^)] = 0.059
*wR*(*F* ^2^) = 0.142
*S* = 0.82
5057 reflections
310 parameters
26 restraints
H atoms treated by a mixture of independent and constrained refinement
Δρ_max_ = 0.66 e Å^−3^
Δρ_min_ = −0.50 e Å^−3^

### 

Data collection: *SMART* (Bruker, 2000 ▶); cell refinement: *SAINT* (Bruker, 2000 ▶); data reduction: *SAINT*; program(s) used to solve structure: *SHELXTL* (Sheldrick, 2008 ▶); program(s) used to refine structure: *SHELXTL*; molecular graphics: *SHELXTL*; software used to prepare material for publication: *SHELXTL* and *publCIF* (Westrip, 2010 ▶).

## Supplementary Material

Crystal structure: contains datablock(s) I, global. DOI: 10.1107/S1600536812007970/tk5060sup1.cif

Structure factors: contains datablock(s) I. DOI: 10.1107/S1600536812007970/tk5060Isup2.hkl

Supplementary material file. DOI: 10.1107/S1600536812007970/tk5060Isup3.mol

Additional supplementary materials:  crystallographic information; 3D view; checkCIF report

## Figures and Tables

**Table 1:** Hydrogen-bond geometry (Å, °)

*D*—H⋯*A*	*D*—H	H⋯*A*	*D*⋯*A*	*D*—H⋯*A*
O1—H26⋯O3^i^	0.86 (1)	1.80 (1)	2.631 (10)	163 (1)
O4—H27⋯O5^ii^	0.86 (1)	2.03 (1)	2.882 (10)	171 (1)
O4—H28⋯O3	0.86 (1)	1.87 (1)	2.684 (10)	158 (5)
O5—H29⋯O11′	0.86 (1)	1.84 (1)	2.695 (10)	174 (1)
O5—H29⋯O11	0.86 (1)	2.09 (1)	2.940 (10)	168 (1)
O5—H30⋯O12′^iii^	0.86 (1)	2.59 (1)	3.162 (10)	125 (1)

Symmetry codes: (i) 
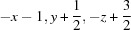
; (ii) 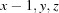; (iii) 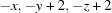.

**Fig. 1.**
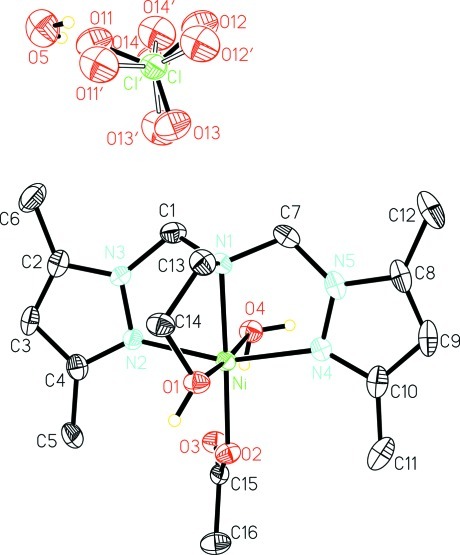
The molecular structure of the title complex with atom labels and 30% probability displacement ellipsoids for non-H atoms. H atoms bound to the C atoms were omitted for clarity.

**Fig. 2.**
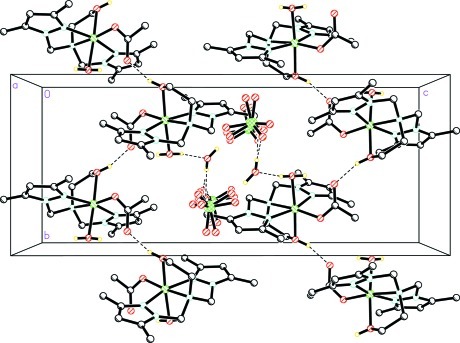
The packing of the complex, viewed approximately down the a axis, showing the O—H···O hydrogen bonds (dashed lines).

## supplementary crystallographic information

### Comment

Zn^II^-bound alkoxides, resulting from the deprotonation of the Zn^II^-coordinated alcoholic hydroxides in Zn^II^-containing enzymes (Lipscomb & Sträter, 1996), usually act as nucleophiles to attack the substrates (*e.g.* phosphates, CO_2_, and carboxy esters). Polyamines with a pendant ethoxyl group can mimic the chemical surroundings of Zn^II^ in the active site of the Zn^II^-containing enzymes (Koike *et al.*, 1995). This encouraged us to investigate the coordination chemistry of transition metal ions with a new ligand containing a N_3_O donor set. In this work, *N*,*N*-bis(3,5-dimethyl-pyrazol-1-yl-methylene)aminoethanol (Malachowski *et al.*, 1992) was reacted with nickel acetate in the presence of sodium perchlorate to yield the title complex as blue crystals in 68% yield. Related structures have been reported previously (Shin *et al.*, 2011; Sundaravel *et al.*, 2011; Xia *et al.*, 2001).

In the structure, the Ni^II^ cation has a six-coordinated geometry consisting of three N atoms and an O atom from the organic ligand, and two O atoms from a water molecule and an acetate (Fig. 1). The Ni—N_pyrazolyl_ bond distances are 2.071 (4) and 2.044 (4) Å, which are shorter than the Ni—N_amino_ bond length (2.124 (3) Å). The Ni—O_acetate_ bond distance is 1.999 (3) Å, which is about 0.1 Å shorter than those of Ni—O_alcohol_ (2.097 (3) Å) and Ni—O_water_ (2.126 (4) Å). The *cis* bond angles are deviate from 90° by about 10°, and the *trans* bond angles deviate from 180° by about 20°. Therefore, the coordination geometry of the Ni^II^ centre is a distorted octahedron. In the crystal, there are O—H···O hydrogen bonds. The unit contents are illustrated in Fig. 2.

### Experimental

A solution of Ni(OAc)_2_.4H_2_O(0.2 mmol) in 2 ml H_2_O was added dropwise to a solution of *N*,*N*-bis(3,5-dimethyl-pyrazol-1-yl-methylene-)aminoethanol (0.2 mmol) in 10 ml of methanol. The blue solution was stirred for 30 min and a drop of saturated NaClO_4_ solution was added to the mixture. The clear solution in a test tube was left undisturbed. Blue crystals were obtained after a week.

### Refinement

H atoms bonded to O atoms were located in a difference map and refined with distance restraints of O—H = 0.86±0.01 Å. Other H atoms were positioned geometrically and refined using a riding model with C—H = 0.93 (aromatic), C—H = 0.97 (CH_2_) and C—H = 0.96 (CH_3_) Å. All H atoms were refined with *U*_iso_(H) = 1.2 (1.5 for methyl groups) *U*_eq_(C). The perchlorate is disordered and refined over two positions. The site occupancy factors of the two positions were refined to a ratio 0.525 (19) and 0.475 (19), and with distances restraints of Cl—O = 1.44 (1) Å.

### Crystal data

**Table:**

[Ni(C_2_H_3_O_2_)(C_14_H_23_N_5_O)(H_2_O)]ClO_4_·H_2_O	*F*(000) = 1112
*M**_r_* = 530.61	*D*_x_ = 1.514 Mg m^−^^3^
Monoclinic, *P*2_1_/*c*	Mo *K*α radiation, λ = 0.71073 Å
Hall symbol: -P 2ybc	Cell parameters from 1110 reflections
*a* = 9.6055 (11) Å	θ = 5.3°
*b* = 9.9889 (11) Å	µ = 1.00 mm^−^^1^
*c* = 24.258 (3) Å	*T* = 293 K
β = 90.284 (2)°	Block, blue
*V* = 2327.5 (5) Å^3^	0.43 × 0.37 × 0.21 mm
*Z* = 4	

### Data collection

**Table:**

Bruker APEX CCD diffractometer	5057 independent reflections
Radiation source: fine-focus sealed tube	2284 reflections with *I* > 2σ(*I*)
Graphite monochromator	*R*_int_ = 0.082
φ and ω scans	θ_max_ = 27.0°, θ_min_ = 1.7°
Absorption correction: empirical (using intensity measurements) (*SADABS*; Sheldrick, 2003)	*h* = −12→12
*T*_min_ = 0.732, *T*_max_ = 1.000	*k* = −9→12
13249 measured reflections	*l* = −30→24

### Refinement

**Table:**

Refinement on *F*^2^	Primary atom site location: structure-invariant direct methods
Least-squares matrix: full	Secondary atom site location: difference Fourier map
*R*[*F*^2^ > 2σ(*F*^2^)] = 0.059	Hydrogen site location: inferred from neighbouring sites
*wR*(*F*^2^) = 0.142	H atoms treated by a mixture of independent and constrained refinement
*S* = 0.82	*w* = 1/[σ^2^(*F*_o_^2^) + (0.0493*P*)^2^] where *P* = (*F*_o_^2^ + 2*F*_c_^2^)/3
5057 reflections	(Δ/σ)_max_ = 0.001
310 parameters	Δρ_max_ = 0.66 e Å^−^^3^
26 restraints	Δρ_min_ = −0.50 e Å^−^^3^

### Special details

**Table:**

Geometry. All e.s.d.'s (except the e.s.d. in the dihedral angle between two l.s. planes) are estimated using the full covariance matrix. The cell e.s.d.'s are taken into account individually in the estimation of e.s.d.'s in distances, angles and torsion angles; correlations between e.s.d.'s in cell parameters are only used when they are defined by crystal symmetry. An approximate (isotropic) treatment of cell e.s.d.'s is used for estimating e.s.d.'s involving l.s. planes.
Refinement. Refinement of *F*^2^ against ALL reflections. The weighted *R*-factor *wR* and goodness of fit *S* are based on *F*^2^, conventional *R*-factors *R* are based on *F*, with *F* set to zero for negative *F*^2^. The threshold expression of *F*^2^ > σ(*F*^2^) is used only for calculating *R*-factors(gt) *etc*. and is not relevant to the choice of reflections for refinement. *R*-factors based on *F*^2^ are statistically about twice as large as those based on *F*, and *R*-factors based on ALL data will be even larger.

### Fractional atomic coordinates and isotropic or equivalent isotropic displacement parameters (Å^2^)

**Table:**

	*x*	*y*	*z*	*U*_iso_*/*U*_eq_	Occ. (<1)
Ni	−0.44003 (7)	1.26474 (6)	0.84018 (3)	0.0315 (2)	
Cl	−0.00311 (17)	0.76885 (16)	0.95183 (7)	0.0575 (4)	0.525 (19)
Cl'	−0.00311 (17)	0.76885 (16)	0.95183 (7)	0.0575 (4)	0.475 (19)
N1	−0.2854 (4)	1.2368 (4)	0.90201 (15)	0.0328 (10)	
N2	−0.2798 (4)	1.1888 (4)	0.79269 (17)	0.0342 (10)	
N3	−0.1741 (4)	1.1411 (4)	0.82475 (17)	0.0355 (10)	
N4	−0.5531 (5)	1.3175 (4)	0.90797 (17)	0.0374 (11)	
N5	−0.4845 (5)	1.2870 (4)	0.95579 (18)	0.0436 (12)	
O1	−0.3473 (4)	1.4547 (3)	0.83710 (15)	0.0383 (9)	
H26	−0.357 (6)	1.491 (5)	0.8051 (10)	0.07 (2)*	
O2	−0.5772 (3)	1.3140 (3)	0.78136 (14)	0.0383 (9)	
O3	−0.6299 (4)	1.1117 (3)	0.74957 (14)	0.0437 (9)	
O4	−0.5166 (4)	1.0662 (4)	0.84915 (19)	0.0434 (9)	
H27	−0.572 (4)	1.067 (5)	0.8766 (14)	0.050 (19)*	
H28	−0.565 (5)	1.062 (6)	0.8195 (14)	0.08 (2)*	
O5	0.2962 (5)	1.0394 (6)	0.9409 (2)	0.0831 (15)	
H29	0.240 (5)	0.972 (4)	0.938 (2)	0.09 (3)*	
H30	0.276 (6)	1.100 (4)	0.965 (2)	0.08 (3)*	
O11	0.1400 (8)	0.7866 (11)	0.9351 (5)	0.088 (3)	0.525 (19)
O11'	0.1098 (13)	0.8400 (14)	0.9279 (5)	0.106 (3)	0.475 (19)
O12	−0.0120 (12)	0.7935 (11)	1.0102 (3)	0.088 (3)	0.525 (19)
O12'	−0.0278 (16)	0.8426 (13)	1.0018 (4)	0.106 (3)	0.475 (19)
O13	−0.1096 (9)	0.8469 (13)	0.9288 (5)	0.088 (3)	0.525 (19)
O13'	−0.1122 (10)	0.7788 (18)	0.9127 (5)	0.106 (3)	0.475 (19)
O14	−0.0262 (12)	0.6290 (7)	0.9444 (5)	0.088 (3)	0.525 (19)
O14'	0.0112 (16)	0.6299 (8)	0.9658 (6)	0.106 (3)	0.475 (19)
C1	−0.1981 (6)	1.1237 (5)	0.8832 (2)	0.0438 (14)	
H1A	−0.1102	1.1230	0.9030	0.053*	
H1B	−0.2452	1.0394	0.8899	0.053*	
C2	−0.0605 (6)	1.1114 (5)	0.7946 (2)	0.0459 (15)	
C3	−0.0943 (6)	1.1418 (5)	0.7411 (2)	0.0482 (16)	
H3A	−0.0374	1.1314	0.7105	0.058*	
C4	−0.2312 (6)	1.1917 (5)	0.7410 (2)	0.0409 (14)	
C5	−0.3171 (6)	1.2402 (5)	0.6948 (2)	0.0535 (16)	
H5A	−0.4067	1.2672	0.7083	0.080*	
H5B	−0.3287	1.1698	0.6683	0.080*	
H5C	−0.2721	1.3152	0.6778	0.080*	
C6	0.0674 (6)	1.0516 (6)	0.8194 (3)	0.072 (2)	
H6A	0.0559	1.0437	0.8585	0.107*	
H6B	0.1458	1.1081	0.8118	0.107*	
H6C	0.0829	0.9646	0.8038	0.107*	
C7	−0.3614 (6)	1.2020 (5)	0.9529 (2)	0.0475 (15)	
H7A	−0.3885	1.1084	0.9522	0.057*	
H7B	−0.3024	1.2166	0.9850	0.057*	
C8	−0.5593 (7)	1.3211 (6)	1.0010 (2)	0.0497 (16)	
C9	−0.6788 (7)	1.3771 (5)	0.9807 (2)	0.0536 (17)	
H9A	−0.7517	1.4113	1.0014	0.064*	
C10	−0.6722 (6)	1.3739 (5)	0.9235 (2)	0.0442 (15)	
C11	−0.7764 (6)	1.4210 (5)	0.8818 (3)	0.0629 (18)	
H11B	−0.7416	1.4047	0.8454	0.094*	
H11C	−0.7923	1.5152	0.8866	0.094*	
H11D	−0.8623	1.3734	0.8867	0.094*	
C12	−0.5118 (7)	1.2947 (6)	1.0577 (2)	0.078 (2)	
H12A	−0.4213	1.2540	1.0569	0.117*	
H12B	−0.5761	1.2354	1.0755	0.117*	
H12C	−0.5070	1.3774	1.0778	0.117*	
C13	−0.2072 (6)	1.3644 (5)	0.9084 (2)	0.0425 (14)	
H13A	−0.1120	1.3446	0.9191	0.051*	
H13B	−0.2490	1.4167	0.9377	0.051*	
C14	−0.2067 (6)	1.4462 (5)	0.8560 (2)	0.0424 (14)	
H14A	−0.1700	1.5350	0.8632	0.051*	
H14B	−0.1491	1.4035	0.8284	0.051*	
C15	−0.6364 (5)	1.2371 (5)	0.7480 (2)	0.0351 (12)	
C16	−0.7201 (6)	1.3043 (5)	0.7039 (2)	0.0552 (16)	
H16A	−0.7145	1.3996	0.7084	0.083*	
H16B	−0.8155	1.2763	0.7064	0.083*	
H16C	−0.6840	1.2800	0.6684	0.083*	

### Atomic displacement parameters (Å^2^)

**Table:**

	*U*^11^	*U*^22^	*U*^33^	*U*^12^	*U*^13^	*U*^23^
Ni	0.0311 (4)	0.0329 (4)	0.0304 (4)	0.0005 (3)	−0.0002 (3)	−0.0012 (3)
Cl	0.0533 (10)	0.0626 (11)	0.0566 (10)	−0.0156 (8)	−0.0060 (8)	0.0043 (9)
Cl'	0.0533 (10)	0.0626 (11)	0.0566 (10)	−0.0156 (8)	−0.0060 (8)	0.0043 (9)
N1	0.037 (3)	0.032 (2)	0.029 (2)	−0.003 (2)	−0.0011 (19)	0.0008 (19)
N2	0.031 (3)	0.039 (2)	0.032 (3)	0.0024 (19)	0.000 (2)	−0.0026 (19)
N3	0.029 (3)	0.041 (3)	0.036 (3)	0.005 (2)	−0.002 (2)	0.001 (2)
N4	0.039 (3)	0.039 (2)	0.034 (3)	−0.001 (2)	0.003 (2)	0.000 (2)
N5	0.051 (3)	0.044 (3)	0.036 (3)	−0.008 (2)	0.012 (2)	−0.003 (2)
O1	0.037 (2)	0.036 (2)	0.042 (2)	−0.0039 (16)	−0.0077 (19)	0.0048 (18)
O2	0.039 (2)	0.036 (2)	0.040 (2)	0.0020 (16)	−0.0108 (18)	−0.0072 (17)
O3	0.052 (3)	0.034 (2)	0.045 (2)	−0.0001 (17)	−0.0064 (19)	−0.0017 (17)
O4	0.042 (3)	0.044 (2)	0.045 (3)	−0.0032 (18)	−0.003 (2)	0.001 (2)
O5	0.084 (4)	0.073 (4)	0.092 (4)	−0.034 (3)	0.006 (3)	0.006 (3)
O11	0.086 (5)	0.067 (4)	0.109 (5)	0.005 (3)	−0.015 (3)	0.000 (3)
O11'	0.118 (7)	0.093 (6)	0.106 (6)	−0.028 (4)	−0.013 (4)	0.003 (4)
O12	0.086 (5)	0.067 (4)	0.109 (5)	0.005 (3)	−0.015 (3)	0.000 (3)
O12'	0.118 (7)	0.093 (6)	0.106 (6)	−0.028 (4)	−0.013 (4)	0.003 (4)
O13	0.086 (5)	0.067 (4)	0.109 (5)	0.005 (3)	−0.015 (3)	0.000 (3)
O13'	0.118 (7)	0.093 (6)	0.106 (6)	−0.028 (4)	−0.013 (4)	0.003 (4)
O14	0.086 (5)	0.067 (4)	0.109 (5)	0.005 (3)	−0.015 (3)	0.000 (3)
O14'	0.118 (7)	0.093 (6)	0.106 (6)	−0.028 (4)	−0.013 (4)	0.003 (4)
C1	0.047 (4)	0.042 (3)	0.043 (4)	0.008 (3)	−0.008 (3)	0.001 (3)
C2	0.036 (4)	0.046 (3)	0.056 (4)	0.000 (3)	0.009 (3)	−0.004 (3)
C3	0.036 (4)	0.052 (4)	0.056 (4)	0.001 (3)	0.019 (3)	−0.006 (3)
C4	0.046 (4)	0.037 (3)	0.040 (4)	−0.004 (3)	0.008 (3)	−0.005 (3)
C5	0.054 (4)	0.078 (4)	0.029 (3)	0.002 (3)	0.007 (3)	0.003 (3)
C6	0.039 (4)	0.081 (5)	0.095 (6)	0.014 (3)	0.005 (4)	0.011 (4)
C7	0.059 (4)	0.052 (4)	0.031 (3)	−0.001 (3)	−0.004 (3)	0.006 (3)
C8	0.059 (5)	0.053 (4)	0.037 (4)	−0.017 (3)	0.018 (3)	−0.009 (3)
C9	0.051 (4)	0.054 (4)	0.055 (4)	−0.012 (3)	0.027 (4)	−0.020 (3)
C10	0.044 (4)	0.030 (3)	0.058 (4)	−0.011 (3)	0.018 (3)	−0.011 (3)
C11	0.039 (4)	0.057 (4)	0.092 (5)	0.008 (3)	0.009 (4)	−0.014 (4)
C12	0.103 (6)	0.096 (5)	0.036 (4)	−0.014 (4)	0.020 (4)	−0.008 (3)
C13	0.046 (4)	0.042 (3)	0.040 (3)	−0.007 (3)	−0.004 (3)	−0.006 (3)
C14	0.044 (4)	0.041 (3)	0.042 (4)	−0.007 (3)	−0.010 (3)	0.003 (3)
C15	0.028 (3)	0.045 (3)	0.033 (3)	0.001 (3)	0.002 (2)	0.002 (3)
C16	0.051 (4)	0.055 (4)	0.059 (4)	0.000 (3)	−0.021 (3)	0.002 (3)

### Geometric parameters (Å, º)

**Table:**

Ni—O2	1.999 (3)	C2—C6	1.490 (7)
Ni—N4	2.044 (4)	C3—C4	1.406 (7)
Ni—N2	2.071 (4)	C3—H3A	0.9300
Ni—O1	2.097 (3)	C4—C5	1.471 (7)
Ni—N1	2.124 (4)	C5—H5A	0.9600
Ni—O4	2.126 (4)	C5—H5B	0.9600
Cl—O13	1.401 (7)	C5—H5C	0.9600
Cl—O14	1.426 (7)	C6—H6A	0.9600
Cl—O12	1.439 (7)	C6—H6B	0.9600
Cl—O11	1.446 (7)	C6—H6C	0.9600
N1—C7	1.480 (6)	C7—H7A	0.9700
N1—C1	1.481 (6)	C7—H7B	0.9700
N1—C13	1.487 (6)	C8—C9	1.368 (8)
N2—C4	1.339 (6)	C8—C12	1.471 (8)
N2—N3	1.362 (5)	C9—C10	1.389 (7)
N3—C2	1.349 (6)	C9—H9A	0.9300
N3—C1	1.447 (6)	C10—C11	1.495 (7)
N4—C10	1.332 (6)	C11—H11B	0.9600
N4—N5	1.366 (5)	C11—H11C	0.9600
N5—C8	1.358 (6)	C11—H11D	0.9600
N5—C7	1.458 (6)	C12—H12A	0.9600
O1—C14	1.427 (6)	C12—H12B	0.9600
O1—H26	0.863 (10)	C12—H12C	0.9600
O2—C15	1.251 (6)	C13—C14	1.512 (6)
O3—C15	1.255 (5)	C13—H13A	0.9700
O4—H27	0.858 (10)	C13—H13B	0.9700
O4—H28	0.856 (10)	C14—H14A	0.9700
O5—H29	0.861 (10)	C14—H14B	0.9700
O5—H30	0.862 (10)	C15—C16	1.494 (7)
C1—H1A	0.9700	C16—H16A	0.9600
C1—H1B	0.9700	C16—H16B	0.9600
C2—C3	1.371 (7)	C16—H16C	0.9600
			
O2—Ni—N4	99.19 (16)	C3—C4—C5	129.7 (5)
O2—Ni—N2	100.50 (15)	C4—C5—H5A	109.5
N4—Ni—N2	160.25 (17)	C4—C5—H5B	109.5
O2—Ni—O1	91.73 (14)	H5A—C5—H5B	109.5
N4—Ni—O1	91.29 (15)	C4—C5—H5C	109.5
N2—Ni—O1	89.70 (15)	H5A—C5—H5C	109.5
O2—Ni—N1	172.99 (14)	H5B—C5—H5C	109.5
N4—Ni—N1	80.69 (16)	C2—C6—H6A	109.5
N2—Ni—N1	79.96 (16)	C2—C6—H6B	109.5
O1—Ni—N1	81.27 (14)	H6A—C6—H6B	109.5
O2—Ni—O4	94.35 (15)	C2—C6—H6C	109.5
N4—Ni—O4	88.45 (16)	H6A—C6—H6C	109.5
N2—Ni—O4	88.49 (16)	H6B—C6—H6C	109.5
O1—Ni—O4	173.88 (16)	N5—C7—N1	107.8 (4)
N1—Ni—O4	92.65 (16)	N5—C7—H7A	110.1
O13—Cl—O14	112.4 (5)	N1—C7—H7A	110.1
O13—Cl—O12	104.5 (6)	N5—C7—H7B	110.1
O14—Cl—O12	106.4 (5)	N1—C7—H7B	110.1
O13—Cl—O11	120.9 (6)	H7A—C7—H7B	108.5
O14—Cl—O11	103.4 (6)	N5—C8—C9	104.9 (5)
O12—Cl—O11	108.4 (7)	N5—C8—C12	123.3 (6)
C7—N1—C1	111.1 (4)	C9—C8—C12	131.9 (6)
C7—N1—C13	111.4 (4)	C8—C9—C10	108.0 (5)
C1—N1—C13	113.6 (4)	C8—C9—H9A	126.0
C7—N1—Ni	105.9 (3)	C10—C9—H9A	126.0
C1—N1—Ni	106.1 (3)	N4—C10—C9	109.6 (6)
C13—N1—Ni	108.2 (3)	N4—C10—C11	121.0 (5)
C4—N2—N3	106.2 (4)	C9—C10—C11	129.4 (5)
C4—N2—Ni	140.9 (4)	C10—C11—H11B	109.5
N3—N2—Ni	111.4 (3)	C10—C11—H11C	109.5
C2—N3—N2	111.7 (4)	H11B—C11—H11C	109.5
C2—N3—C1	129.6 (5)	C10—C11—H11D	109.5
N2—N3—C1	118.6 (4)	H11B—C11—H11D	109.5
C10—N4—N5	105.4 (4)	H11C—C11—H11D	109.5
C10—N4—Ni	142.9 (4)	C8—C12—H12A	109.5
N5—N4—Ni	111.7 (3)	C8—C12—H12B	109.5
C8—N5—N4	112.1 (5)	H12A—C12—H12B	109.5
C8—N5—C7	128.1 (5)	C8—C12—H12C	109.5
N4—N5—C7	118.5 (4)	H12A—C12—H12C	109.5
C14—O1—Ni	109.7 (3)	H12B—C12—H12C	109.5
C14—O1—H26	114 (4)	N1—C13—C14	112.3 (4)
Ni—O1—H26	112 (4)	N1—C13—H13A	109.2
C15—O2—Ni	127.3 (3)	C14—C13—H13A	109.2
Ni—O4—H27	107 (3)	N1—C13—H13B	109.2
Ni—O4—H28	99 (4)	C14—C13—H13B	109.2
H27—O4—H28	108 (5)	H13A—C13—H13B	107.9
H29—O5—H30	117 (3)	O1—C14—C13	107.2 (4)
N3—C1—N1	107.7 (4)	O1—C14—H14A	110.3
N3—C1—H1A	110.2	C13—C14—H14A	110.3
N1—C1—H1A	110.2	O1—C14—H14B	110.3
N3—C1—H1B	110.2	C13—C14—H14B	110.3
N1—C1—H1B	110.2	H14A—C14—H14B	108.5
H1A—C1—H1B	108.5	O2—C15—O3	124.8 (5)
N3—C2—C3	106.0 (5)	O2—C15—C16	115.4 (5)
N3—C2—C6	122.5 (5)	O3—C15—C16	119.8 (5)
C3—C2—C6	131.5 (5)	C15—C16—H16A	109.5
C2—C3—C4	107.3 (5)	C15—C16—H16B	109.5
C2—C3—H3A	126.4	H16A—C16—H16B	109.5
C4—C3—H3A	126.4	C15—C16—H16C	109.5
N2—C4—C3	108.8 (5)	H16A—C16—H16C	109.5
N2—C4—C5	121.5 (5)	H16B—C16—H16C	109.5
			
O2—Ni—N1—C7	118.3 (11)	O4—Ni—O1—C14	18.1 (16)
N4—Ni—N1—C7	28.7 (3)	N4—Ni—O2—C15	−115.7 (4)
N2—Ni—N1—C7	−147.3 (3)	N2—Ni—O2—C15	62.7 (4)
O1—Ni—N1—C7	121.4 (3)	O1—Ni—O2—C15	152.7 (4)
O4—Ni—N1—C7	−59.3 (3)	N1—Ni—O2—C15	155.8 (11)
O2—Ni—N1—C1	−123.5 (11)	O4—Ni—O2—C15	−26.6 (4)
N4—Ni—N1—C1	146.9 (3)	C2—N3—C1—N1	145.0 (5)
N2—Ni—N1—C1	−29.1 (3)	N2—N3—C1—N1	−38.1 (6)
O1—Ni—N1—C1	−120.4 (3)	C7—N1—C1—N3	157.0 (4)
O4—Ni—N1—C1	58.9 (3)	C13—N1—C1—N3	−76.5 (5)
O2—Ni—N1—C13	−1.3 (13)	Ni—N1—C1—N3	42.3 (4)
N4—Ni—N1—C13	−90.9 (3)	N2—N3—C2—C3	0.3 (6)
N2—Ni—N1—C13	93.1 (3)	C1—N3—C2—C3	177.3 (5)
O1—Ni—N1—C13	1.9 (3)	N2—N3—C2—C6	−177.1 (5)
O4—Ni—N1—C13	−178.9 (3)	C1—N3—C2—C6	−0.1 (8)
O2—Ni—N2—C4	20.1 (6)	N3—C2—C3—C4	0.6 (6)
N4—Ni—N2—C4	−164.6 (5)	C6—C2—C3—C4	177.7 (6)
O1—Ni—N2—C4	−71.6 (5)	N3—N2—C4—C3	1.4 (5)
N1—Ni—N2—C4	−152.8 (6)	Ni—N2—C4—C3	165.0 (4)
O4—Ni—N2—C4	114.2 (5)	N3—N2—C4—C5	−179.1 (4)
O2—Ni—N2—N3	−176.8 (3)	Ni—N2—C4—C5	−15.5 (8)
N4—Ni—N2—N3	−1.5 (6)	C2—C3—C4—N2	−1.3 (6)
O1—Ni—N2—N3	91.5 (3)	C2—C3—C4—C5	179.3 (5)
N1—Ni—N2—N3	10.3 (3)	C8—N5—C7—N1	−157.5 (5)
O4—Ni—N2—N3	−82.7 (3)	N4—N5—C7—N1	36.8 (6)
C4—N2—N3—C2	−1.1 (5)	C1—N1—C7—N5	−155.8 (4)
Ni—N2—N3—C2	−170.1 (3)	C13—N1—C7—N5	76.5 (5)
C4—N2—N3—C1	−178.5 (4)	Ni—N1—C7—N5	−41.0 (4)
Ni—N2—N3—C1	12.5 (5)	N4—N5—C8—C9	−0.9 (6)
O2—Ni—N4—C10	−4.3 (6)	C7—N5—C8—C9	−167.4 (5)
N2—Ni—N4—C10	−179.7 (5)	N4—N5—C8—C12	177.7 (5)
O1—Ni—N4—C10	87.6 (6)	C7—N5—C8—C12	11.2 (8)
N1—Ni—N4—C10	168.6 (6)	N5—C8—C9—C10	0.4 (6)
O4—Ni—N4—C10	−98.5 (6)	C12—C8—C9—C10	−178.0 (6)
O2—Ni—N4—N5	176.8 (3)	N5—N4—C10—C9	−0.7 (5)
N2—Ni—N4—N5	1.4 (6)	Ni—N4—C10—C9	−179.7 (4)
O1—Ni—N4—N5	−91.3 (3)	N5—N4—C10—C11	−179.9 (4)
N1—Ni—N4—N5	−10.3 (3)	Ni—N4—C10—C11	1.2 (8)
O4—Ni—N4—N5	82.6 (3)	C8—C9—C10—N4	0.2 (6)
C10—N4—N5—C8	1.0 (5)	C8—C9—C10—C11	179.2 (5)
Ni—N4—N5—C8	−179.6 (3)	C7—N1—C13—C14	−143.8 (4)
C10—N4—N5—C7	169.0 (4)	C1—N1—C13—C14	89.8 (5)
Ni—N4—N5—C7	−11.7 (5)	Ni—N1—C13—C14	−27.7 (5)
O2—Ni—O1—C14	−155.1 (3)	Ni—O1—C14—C13	−46.3 (4)
N4—Ni—O1—C14	105.7 (3)	N1—C13—C14—O1	49.7 (5)
N2—Ni—O1—C14	−54.6 (3)	Ni—O2—C15—O3	7.7 (7)
N1—Ni—O1—C14	25.3 (3)	Ni—O2—C15—C16	−172.5 (3)

### Hydrogen-bond geometry (Å, º)

**Table:**

*D*—H···*A*	*D*—H	H···*A*	*D*···*A*	*D*—H···*A*
O1—H26···O3^i^	0.86 (1)	1.80 (1)	2.631 (10)	163 (1)
O4—H27···O5^ii^	0.86 (1)	2.03 (1)	2.882 (10)	171 (1)
O4—H28···O3	0.86 (1)	1.87 (1)	2.684 (10)	158 (5)
O5—H29···O11′	0.86 (1)	1.84 (1)	2.695 (10)	174 (1)
O5—H29···O11	0.86 (1)	2.09 (1)	2.940 (10)	168 (1)
O5—H30···O12′^iii^	0.86 (1)	2.59 (1)	3.162 (10)	125 (1)

Symmetry codes: (i) −*x*−1, *y*+1/2, −*z*+3/2; (ii) *x*−1, *y*, *z*; (iii) −*x*, −*y*+2, −*z*+2.
